# Nursing team proxemic mapping in the hemotherapy space*

**DOI:** 10.1590/1980-220X-REEUSP-2024-0131en

**Published:** 2025-01-27

**Authors:** Francisco Gleidson de Azevedo Gonçalves, Silvia Teresa Carvalho de Araújo, Albert Lengruber de Azevedo, Priscila Brigolini Porfírio Ferreira, Danelia Gómez Torres, Fernanda de Nazaré Almeida Costa, Ariane da Silva Pires, Soraia do Socorro Furtado Bastos

**Affiliations:** 1 Universidade Federal do Rio de Janeiro, Escola de Enfermagem Anna Nery, Departamento de Enfermagem Médico-Cirúrgica, Rio de Janeiro, RJ, Brazil.; 2 Universidade Federal de Roraima, Centro de Ciências da Saúde, Boa Vista, Roraima, Brazil.; 3 Universidad Autonoma del Estado de México, Facultad de Enfermería, Departamento de Enfermería Médico-Quirúrgica, Toluca, Estado de México, México.; 4 Universidade do Estado do Rio de Janeiro, Faculdade de Enfermagem, Departamento de Enfermagem Médico-Cirúrgica, Rio de Janeiro, RJ, Brazil.

**Keywords:** Spatial Behavior, Patient Care Team, Nursing Care, Nonverbal Communication, Hemotherapy Service, Blood Transfusion, Conducta Espacial, Grupo de Atención al Paciente, Atención de Enfermería, Comunicación no Verbal, Servicio de Hemoterapia, Transfusión Sanguínea

## Abstract

**Objective:**

To analyze the influence of proxemic factors on communication and care provided by nursing professionals during transfusion in hemotherapy.

**Method:**

A descriptive, exploratory and qualitative study with 25 nursing professionals from a hospital specializing in onco-hematological diseases in Rio de Janeiro, based on a systematized script, individual records of proxemic factors described by Edward Hall and recorded situational interviews. The analysis considered data thematic content and used the SketchUp 3D Modeling Software Review program to visually demonstrate the behavioral mapping of the interaction of nursing professionals with patients during care.

**Results:**

Intimate, personal, social and public distances translated into proxemic factors such as repeated movements of shoulders, neck and head, which positively influenced nursing care in hemotherapy, favoring the identification of non-verbal and verbal manifestations of the team in interaction with patients.

**Conclusion:**

Nonverbal proxemic communication must result from awareness of the layout of physical space, immediate care actions, pertinent and appropriate proximity, continuous visual surveillance and clear recognition of expressions translated as interest, respect and zeal.

## RESUMEN

**Objetivo:**

Analizar la influencia de los factores proxémicos en la comunicación y el cuidado brindado por los profesionales de enfermería durante el acto transfusional en hemoterapia.

**Método:**

Estudio descriptivo, exploratorio y cualitativo, con 25 profesionales de enfermería de un hospital especializado en enfermedades oncohematológicas de Río de Janeiro, a partir de un guión sistematizado, registro individual de los factores proxémicos descritos por Edward Hall y entrevista situacional grabada. El análisis consideró el contenido temático de los datos y utilizó el programa SketchUp 3D Modeling Software Review para demostrar visualmente el mapeo conductual de la interacción de los profesionales de enfermería con los clientes durante la atención.

**Resultados:**

Las distancias íntimas, personales, sociales y públicas tradujeron factores proxémicos, como movimientos repetidos de hombros, cuello y cabeza, que influyeron positivamente en los cuidados de enfermería en hemoterapia, favoreciendo la identificación de manifestaciones verbales y no verbales del equipo en interacción con el cliente.

**Conclusión:**

La comunicación no verbal proxémica debe resultar de la conciencia de la disposición del espacio físico, de las acciones de cuidado inmediato, de la proximidad pertinente y adecuada, de la vigilancia visual continua y del reconocimiento claro de las expresiones traducidas por interés, respeto y celo.

## INTRODUCTION

Proxemic communication refers to the social study of human beings’ personal space in the interactional field, being determined by the distances that people maintain between themselves^(1)^. Its recognition can occur through vision, hearing, smell and touch, in addition to varying greatly between cultures and processes inherent to the construction of man, acculturation^(1)^, situations and phenomena such as perception, fixed, semi-fixed and informal characteristics of space and reactions and changes in behavior^(2)^.

Fixed characteristics concern buildings, walls, steel, wood, concrete structures, bridges and the organization of cities; ­semi-fixed characteristics concern the arrangement of furniture in a room, the use of ornaments or any behavior of individuals that may suggest acceptance, rejection and escape, whose characteristics may or may not facilitate social relationships; and informal characteristics are created by individuals to favor the establishment of different interactions, such as intimate distance, which does not exceed 45 cm, personal distance, from 46 cm to 120 cm, social distance, from 120 cm to 370 cm, and public distance, above 370 cm^(1)^.

In nursing care, being close, maintaining interest, readiness and bonding can, intentionally or not, express skills and competencies that influence the relationship with the other. Even though proxemic communication seems to be a barrier for many professionals, expressions of pain, frowning, turning the face or even a moan can indicate the need to act in personal and territorial space, institute a continuous assessment, think about verbal and nonverbal communication^(3)^, and discuss, or even develop, an intervention plan that considers the space where interlocutors are present^(4)^.

In settings such as hemotherapy, unlike others occupied by nursing professionals, care tends to be developed from two perspectives: direct care, which requires donor participation, data collection, the moment of donation, the transfusion act, or even a transplant of live blood cells; and indirect care, which applies to the blood component bag and involves identification, storage, fractionation, testing, transportation and its proper disposal^(5,6)^.

Considering that communication must be understood dynamically, direct care, more precisely the act of transfusion, requires constant proximity between people, attentive and qualified listening and looking, as it involves carrying out complementary and complex procedures, such as medical prescription, blood component installation in recipients and its monitoring, identification of critical points, such as sample collection, medical decision to transfuse or not, as well as blood component administration at the bedside, therefore having a beginning, middle and end^(7,8)^.

The experience gained in research related to bodily senses with the aim of understanding proxemic communication, the search for factors that influence and/or interfere with individual perception, the investigation of behaviors and interaction with patients and health team^(2,3)^, as well as knowledge gaps in hemotherapy on behavioral mapping, contributed to delimit the following guiding question: what proxemic factors determine nursing professionals’ communication during transfusion? Thus, the proposed objective was to analyze the influence of proxemic factors on the communication and care provided by nursing professionals during transfusion in hemotherapy.

## METHOD

### Study Design

This is a qualitative, exploratory, descriptive study, anchored in Edward T. Hall’s theoretical framework on proxemic communication and behavioral mapping^(1)^.

### Study Setting

The setting was a blood center linked to the state health department, located in southeastern Brazil. The choice was made because this is a specialized service, a reference in hematology and hemotherapy, which operates every day of the week, 24 hours a day, in outpatient and hospital settings, whose mission is to offer quality care to the population.

### Participants and Selection Criteria

Twenty-five nursing professionals from the hemotherapy service, who had at least six months of experience and were part of the daytime nursing staff roster participated in the study. Those who did not participate in both data collection moments (observation and interview) and who were unable to provide care at the time of data collection (license or medical restriction) were excluded.

### Data Collection Procedure

To collect data, initially, a visit was made to the service and contact was made with nursing management and their respective sector heads, at which time it was clarified that conducting the research would ensure uninterrupted assistance to patients and the guarantee that the researcher’s relationship with participants would be focused on the actions and observations of proxemic behavior.

As agreed, the researcher was inserted and familiarized with the service through an in-person meeting with nursing professionals, privately, through a rotation system, from 7:00 a.m. to 7:30 a.m. This was followed by an explanation of the study method and the items considered in a non-participant observation script. The selection made by the researcher considered the convenience technique, interest and availability of each participant.

Data collection took place between April and December 2022, when nurses and nursing technicians were first invited to participate in the research, followed by reading and subsequently signing the Informed Consent Form. Data collection was carried out by the main researcher and by a nurse who has extensive knowledge in qualitative research in hematology and hemotherapy, with an emphasis on verbal and nonverbal communication during transfusion. To this end, a non-participant observation script was used as an instrument.

Upon acceptance, observations of nursing professionals’ communication during transfusion were carried out. Non-participant observation was carried out with the aim of verifying nursing teams’ behavioral mapping during transfusion care. A script was used, created from the eight proxemic factors proposed by Hall’s theory, such as postural-sex identifiers, sociofugal-sociopetal orientation factors, kinesthetic factors, touching factors, visual factors, thermal factors, olfaction factors, vocal factors. This stage lasted four hours per day and occurred for 90 days, totaling 360 hours.

Subsequently, a semi-structured interview was conducted, consisting of two questions: what precautions were taken during the transfusion procedure? What precautions were taken regarding intimate, personal, social and public distances? The questions were asked at the institution studied, subject to participants’ availability. The researchers recorded the interviews individually in audio, with an average duration of 45 minutes.

The next moment, which lasted a little over 360 hours, was the researcher’s seclusion, when observations recorded in field diary and interviews were transcribed as well as the material produced was read and re-read, which made it possible to understand the interaction between nursing professionals and patients during transfusion and the distances maintained in care.

The COnsolidated criteria for REporting Qualitative research (COREQ)^(9)^ were considered to maintain the rigor of the data organization process.

### Data Analysis and Treatment

The data were organized and prepared for the thematic content analysis modality^(10)^: comprehensive and detailed reading; material analysis; treatment, comprehension and interpretation. Initially, the transcribed material was carefully read, focusing on professionals’ perceptions about the phenomenon under investigation. Then, the content of the speeches that presented similarities and differences was examined and grouped into categories according to the research objectives. To ensure data reliability, the main researcher transcribed the interviews, which were reviewed by a second researcher. Moreover, double coding and error checking were performed to ensure coding tree creation legitimacy.

Subsequently, the generated codes were grouped by similarities and differences in axial coding, also undergoing a refinement in the analysis that gave rise to the central category entitled “Distances and proximities translated by bodily senses in hemotherapy”.

### Ethical Aspects

This study complied with national and international standards of ethics in research involving human beings and was approved in 2020 by the Research Ethics Committee, under Opinion 4,376,390. The research complies with Resolution 466 of December 12, 2012, and the Informed Consent Form was used as the consent instrument. Participant anonymity was guaranteed, and their statements were identified as Nur. Tech. for “Nursing Technician” and Nur. for “Nurse”, followed by the alphanumeric order (1, 2, 3, up to 18) according to the final code presented (Nur. Tech.1, Nur. Tech.2, Nur. Tech.3 up to Nur. Tech.18) and (Nur.1, Nur.2, Nur.3 up to Nur.7).

## RESULTS

Study participants were seven nurses and 18 nursing technicians (n = 25), mostly women (n = 22), married (n = 18), aged between 30 and 45 years (n = 18) and between 45 and 55 years (n = 7), with more than 13 years of experience in the hemotherapy service.

Considering nursing teams occupation in the physical space of hemotherapy, the following proxemic factors were identified: repeated movements of the shoulders, neck and head.

**Figure 1:**
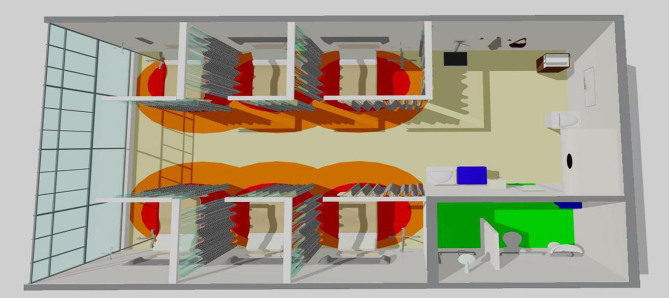
General ward graphical representation. Rio de Janeiro, Brazil, 2023.

**Figure 2:**
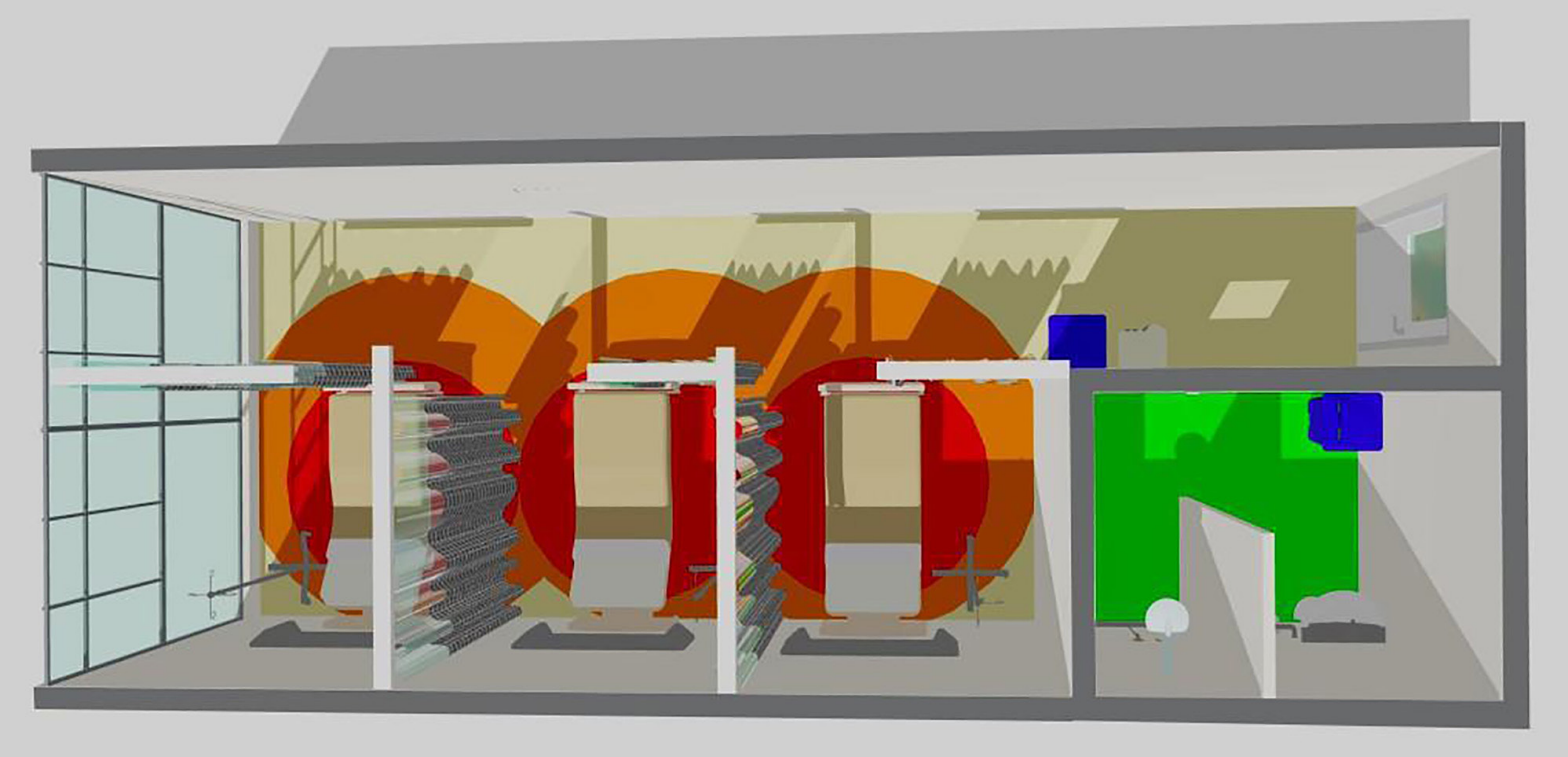
Precautionary ward graphical representation. Rio de Janeiro, Brazil, 2023.

Using the Sketch Up 3D Modeling Software Review program, a graphic representation to visually illustrate, using colors, and behavioral mapping resulting from observations made in the general and precautionary wards were created. In this graphic, red symbolizes intimate distance, which is approximately 45 cm long and ranges from direct physical contact between professionals and patients. The orange hue ranges from 45 cm to 1.20 m, indicating the personal distance maintained between individuals. The yellow hue, which ranges from 1.20 m to 3.60 m, symbolizes social distance. On the other hand, green extends from 3.60 m to the limits of vision or hearing, defining public distance, as represented in Figures 1 and 2.

**Figure 3:**
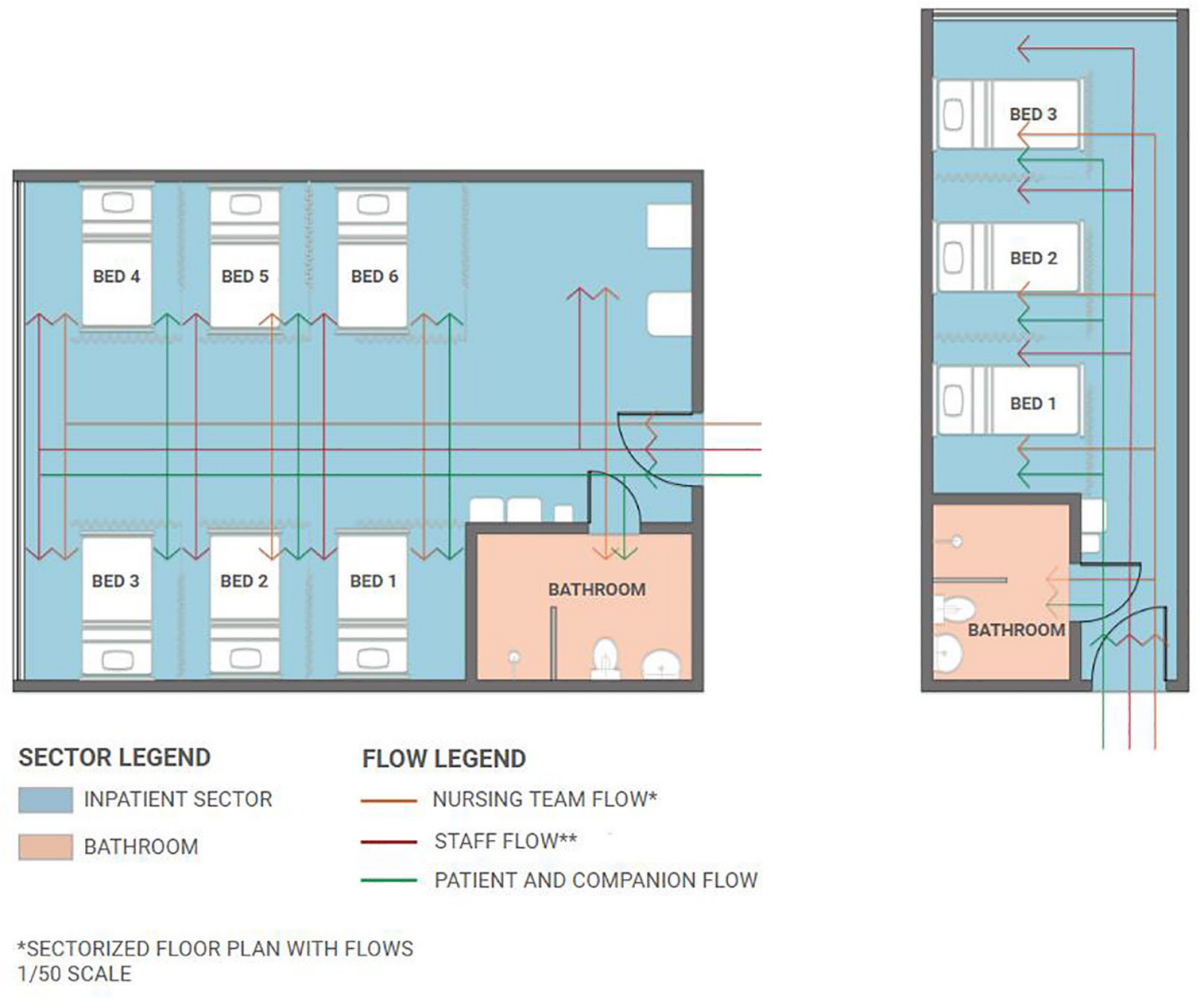
Hemotherapy space flow graphical representation. Rio de Janeiro, Brazil, 2023.

In this environment, there are two wards for the hospitalization of patients with hematological deviations, one of which has six beds and the other has three, aiming to assist those with some type of precaution. Each of these wards is separated by curtains and has an automated bed, an accessory table, a bell - in case there is a need to request the presence of a professional - and a screen. Figure 3 shows the hemotherapy space flow observed at the time of data collection.

The nursing station, in turn, is far from patients’ beds, which requires constant movement of nursing teams. Communication between them almost always occurred through a bell panel that, when activated by patients, indicated the need for a professional from the team. Among the furniture and supplies present in this environment, chairs, tables, benches and daily consumables were observed.

The standing position was mentioned by participants during all care related to blood component bag infusion and removal, i.e., from the verification of personal data such as name, registration, blood type and RH factor to peripheral venipuncture, peripheral venous access removal as well as at hospital discharge, as described:

*(...) I stand up every time I administer premedication to avoid a previous transfusion reaction; when I am asked to clarify doubts; when the blood component bag stops dripping; when it is necessary to puncture a new access; to visualize the bag; when I have to handle a deep venous catheter in the femoral vein (Nur. Tech. 03).*

The sitting position was identified in two moments. The first moment was during the execution of bureaucratic care, such as performing nursing developments, answering phone calls, checking prescriptions, among others. The second moment was when performing some type of sensitive care, such as approaching patients to talk or even clarify any doubts about the transfusion process, as shared:

*(...) I sit down when I’m doing the nursing progress, or when a patient asks why the bag is taking so long; some of them like to talk, and in that case, I stop everything and pull up the chair to give more attention (...) (Nur. Tech. 07).*

*When I’m at the nursing station monitoring the bag, answering the phone to check floor requests, medical prescriptions; checking everything and recording it in the book, on the transfusion map (Nur. 01).*

Regarding the distances maintained between nursing teams and patients, intimate distance stood out. It was ­highlighted by participants during blood component bag installation, checking of vital signs, performing peripheral venipuncture, removing accesses, and/or even at hospital discharge, as indicated:

*I approach every time I go to check patients’ vital signs, their temperature to install a red blood cell transfusion, a plasma or even a platelet concentrate, regardless of what the component is (Nur. Tech. 02).*

*(...) I get closer to patients when I have to connect the blood bag; when I talk about what they might feel when faced with a reaction; or even to check if they have had a previous reaction (Nur. Tech. 14).*

Personal distance was presented by participants when it was necessary to verify patients’ personal data, check their registration number, blood type, RH factor and also during a conversation with patients. It is worth noting that this is a distance at which interlocutors can touch each other or even maintain a closer dialogue without touching.

*I arrive saying “good morning” and asking if they are okay; I try to check how they are; how is the peripheral venous access; if infusion is not flowing well; I check if they have had any previous reaction to the transfusion, the blood type, the RH factor; when I identify myself to patients (Nur. 07).*

*(...) when I approach a patient, the first thing I do is ask their name; introduce myself; I gather their history of reactions, the hemotherapy data itself, such as reactions prior to transfusion; and, if I am called, I visualize the bag; I inspect everything that is necessary (Nur. Tech. 18).*

Social distancing was declared by participants in situations such as the first time a patient received a bag of blood components, when they rang the bell all the time, or even when they requested the presence of a professional because of the fear they felt, as shared:

*(...) there are patients who are very concerned, nervous and anxious about the installation of platelet concentrate, especially those who are having their first or second transfusion. They ask us to stay until they feel safe. In these cases, I try to get closer to patients during transfusion; I pay attention, look at their face, and see how they are behaving, especially if transfusion is flowing (Nur. 06).*

*(...) I approach them every time a patient calls me (...) I make them as comfortable as possible; I talk to them; I give them as much attention as possible; I ask if everything is okay; I make myself available to talk to them (Nur. Tech. 13).*

Public distance was described by participants in situations such as responding to patient requests, adjusting coagulation factor dosages, collecting orders from other hemotherapy services and/or seeking a prescription for premedication with a hemotherapist, according to the following statements:

*(...) when I’m leaving work, a patient calls me, asks for something, information, which is more common. I always approach them, even if I’m not physically close (Nur. Tech. 05).*

*(...) when a patient is undergoing transfusion, I have to go there to check how they are, how the process is progressing; I also have to go to remove the bag, and check the vital signs every time it finishes (Nur. 04).*

## DISCUSSION

Concerning participant description and characterization, it is important to note that all nurses were specialists in hemotherapy. On the other hand, only three professionals from the nursing technical team had such specialization. Their practices occur in accordance with the laws, ordinances and resolutions of the collegiate board of the Brazilian National Health Regulatory Agency and the Ministry of Health, with specific technical procedures that supervise and guide nurses’ and nursing technicians’ activities.

Experienced and conscientious professionals, attentive to postures, behaviors and attitudes, physical space, caring actions, intense proximity, continuous visual surveillance and recognition of expressions of interest, respect and concern, perceive proxemic nonverbal communication. Research indicates that male physicians have loud voices because they are further away from patients and spend more time reading patient records, while female physicians have more satisfied patients because of their less expansive body postures, softer voices and self-touch, conveying a more attentive and medical atmosphere^(4)^.

Participant observation and analysis of the interviews allowed us to identify the behavioral mapping of the colors that represent the distance between the forms of care provided during transfusions. Red symbolizes intimate separation of approximately 45 cm between professionals and patients. The orange scale ranges from 45 cm to 1.20 m, representing the distance that individuals maintain between themselves. Social distance is represented by the yellow measurement, which varies from 1.20 m to 3.60 m. The green shade symbolizes the public extension of 3.60 m up to the limits of vision or hearing^(1)^.

During the checking of vital signs, peripheral venipuncture, blood component bag installation, access line removal and even at hospital discharge, for instance, red indicates intimate distance. Patient personal data, such as registration, blood type and RH factor, are represented by orange to demonstrate personal distance. Yellow symbolizes social distancing, which is seen in circumstances such as the first time a patient receives a blood component bag, in which they often ring the bell or even requests the presence of a professional out of fear. Green represents social distancing, which can be used in situations such as responding to patient requests, adjusting coagulation factor dosages or even receiving requests for other blood therapy services.

When measurable distances in the relational process are recognized, proxemic communication tends to become an additional tool for nursing and health professionals to complement care, in order to value interpersonal relationships and promote health and well-being^(1)^. Thus, in the communicational dimension, the use of space is represented by a distance in the moments of care practice in hemotherapy.

When observing proxemic language, interpersonal relationships and the distance maintained in interaction^(3,11)^, it is clear that intimate space is adopted by most participants, represented by a face-to-face posture, which directs the gaze towards patients, observing, guiding and facilitating the interactional relationship. This relationship can be understood from the set of observations that individuals make of the space and how they use and interpret it within the communicative process. Proxemic language, on the other hand, is influenced by cultural norms, context, spatial obstacles, relationships between interlocutors and the degree of affinity between them^(12)^.

In the context of hemotherapy, proxemic communication represents a powerful tool that facilitates the process of interaction between subjects, but it needs to be constantly explored^(1)^. This requires training and willingness. Close observation of nonverbal signals allows for a reliable perception of the message^(11)^. To interpret it, one needs to know how to deal with their own way of communicating. Its effectiveness increases when you are aware of the importance of body language, especially proximity, posture and eye contact^(11)^.

Investigating behaviors in a highly complex hospital setting for hemotherapy is a difficult process, since care is sometimes directed to blood components and sometimes to patients, which can make its operationalization fragile. Furthermore, attitudes and gestures are often difficult to identify and categorize, since customer service with blood products is long and, depending on the condition, constant, becoming a lifelong habit^(13)^.

The behavior map created from the distance zones was used to relate the distances between individuals, while the colors were used to characterize the proxemic characteristics of the nursing teams involved in hemotherapy procedures. This makes it possible to increase the possibility of observing and detecting patients’ verbal or nonverbal signals, contributing to more effective communication and interpersonal relationships between the subjects of interaction, since it allows analyzing and modifying certain behaviors or postures in approaches and personal areas^(1,2)^.

In this scenario, intimate distance is most evident during blood component installation, when preparing the skin for venipuncture and when removing access points. The care provided requires maximum proximity between hemotherapy nursing professionals and patients, whose bonds can be strengthened, given that interactions occur continuously, uninterruptedly, during the 24-hour shift, month after month, year after year, resulting in an intense closeness due to length of stay and prolonged treatment^(11,12)^.

Personal distance occurs when providers approach patients from a distance, ask their name, introduce themselves, identify history of previous transfusion reactions, view the bag, and inspect the transfusion^(13)^.

In relation to social distance, interactions are determined by the bond established between people, by the existing feeling and by the form of care at that moment. In hemotherapy, communication between nurses and patients stands out, every day, by proxemics, whether at the time of observing the transfusion act from a distance, with a more acute look in search of transfusion reactions, or during the waiting period of the first ten minutes after installing the blood component, sitting at the nursing station^(14,15)^.

In public distance, vision is impaired, as is the sharpness of images. The tone of voice is raised, which can be compared to an escape behavior^(14,16)^. Therefore, it is a great challenge to identify methods that allow us to identify proxemic behaviors that manifest themselves in the daily reality of nursing teams. Nonverbal signals provide clues about people’s interaction with their environments, which could not be obtained by other research methods^(1,14,16)^.

In this investigation, the public distance changed to intimate distance when professionals were attentive, in the first minutes of transfusion, and when patients said goodbye from a distance. Thus, the public distance is established throughout the transfusion process and the intimate distance is established at the end of it, with the checking of vital signs after transfusion.

It is important to emphasize that the context in which people communicate often influences the proximity or distance between bodies and that both the regularity and content of messages are affected by various elements of the environment^(1)^. This environment affects behavior, but it can also change it and provoke certain reactions. When one is aware of the location, one can purposefully use it to achieve the desired responses. It is clear that proximity makes it possible to obtain more information about the other individual^(1)^.

The arrangement of fixed and semi-fixed spaces occupied by patients impacts professionals’ proxemic behavior and their development, especially when there is, *a priori*, knowledge and awareness of the concept and perceptive influence.

Therefore, the size and arrangement of objects and furniture, appropriate lighting, ceiling color and room temperature affect the path and visual and physical interaction between individuals. If a more accessible environment can increase the frequency of interactions, small changes in space can also facilitate and favor the proxemics of approach^(1,17)^, expanding access and interaction.

Thus, attention to these elements affects communication, defining skills and competencies in interaction with others^(18)^. Maintaining proximity, maintaining interest, availability and connection facilitates the expression of emotions, needs and assessment during interaction^(19,20)^.

Therefore, as a health-related profession, nursing must also be concerned with the pattern of proxemic communication in the care environment in the context of hemotherapy^(18)^. The ability to provide patients with the chance for a more dignified and understandable life requires knowledge of body language and an understanding of their position in the therapeutic context^(2,16)^.

It is important to highlight that care actions were predominantly marked by professionals’ gestures in intimate, personal, social and public environments. In addition to this, the verbalization of nursing teams in hemotherapy caused variations in the use and extension of the body’s senses. Therefore, in order to be able to think, debate, create and implement intervention strategies for patients, it is crucial that nursing teams in hemotherapy first understand the space as one of the elements that surround interlocutors, which can occasionally influence the degree and type of interaction that occurs^(18,21)^.

Since this is a topic that addresses proxemic communication as a form of nonverbal communication, the study was limited by COVID-19. It was not possible to verify all proxemics in their entirety, since the use of masks, necessary during the pandemic, influenced some observations of participants’ facial and body expressions during interactions. In this regard, the use of this protective equipment brought some difficulties in interaction, such as embarrassment in communicating orally and difficulty in perceiving facial expressions. Patients and nurses were unable to communicate well and spoke louder, in addition to not establishing lip reading and recognition of other visual cues on the face.

It is recommended that multicenter studies be conducted in other regions of the country and in other blood centers that still lack such data as well as quantitative studies with that could better elucidate the distances and proximities in the relationships between patient and professional during nursing care. In addition, there is little research in Brazil that focuses on proxemic communication in hemotherapy.

It is believed that the results of this study can contribute to improving the analysis of nurses’ proxemic behavior, as well as for other blood centers and institutions, to consider incorporating these findings to qualify and benefit patients.

## CONCLUSION

The results reveal that proxemic nonverbal communication is influenced by physical space, care actions, proximity, continuous visual surveillance, recognition of facial expressions, respect and care. Consequently, in order to provide quality care, it is essential that professionals are attentive to postures, behaviors and attitudes of their patients. This type of communication requires constant attention, qualified listening and attentive looks, and is a process that must be understood dynamically.

The identification of nonverbal and verbal expressions of hemotherapy nursing teams in their interactions with patients was facilitated by behavioral mapping, which used the body’s senses to identify nonverbal signals and expressions. It made it possible to verify how both participants may or may not benefit from interactions and care within the hemotherapy context. The context of hemotherapy is unique, and nursing professionals’ behavior requires the development of a tool to assess interactions in care.

It was found that proxemic behavior is influenced by the physical space and that the measures taken by professionals in hemotherapy influence the mapping observed in interactions and care provided to patients. In the study, nursing teams in hemotherapy demonstrated a good bond with patients, demonstrating interest, respect and care, evidenced by intense proximity, through constant visual monitoring, especially after blood component installation.

Proxemic behavior, which is part of nonverbal language, is often mysterious in the communication process. Typically, interactions of health teams with patients during hemotherapy occurred at all distances suggested by Hall, with a predominance of intimate distance, followed by personal distance, social distance and public distance, all established during nursing care in hemotherapy. The findings highlighted that proxemic behavior and behavioral analysis helped to identify nonverbal and verbal expressions. Furthermore, the interaction of hemotherapy nursing teams with patients influenced the use of body senses to identify nonverbal signals and expressions.

There are still initial studies on proxemic communication between nursing teams and patients during hemotherapy. Therefore, it is considered that this study can serve as a starting point for future research, especially to implement team actions and (re)examine communication with this audience.
