# Supplementary material for: Nursing team proxemic mapping in the hemotherapy space
Source: Rev Esc Enferm USP. 2025 Jan 27;58:e20240131. doi: 10.1590/1980-220X-REEUSP-2024-0131en (PMC11773683; doi:10.1590/1980-220X-REEUSP-2024-0131en)
Supplement: Translation

# Translation: Mapeamento proxêmico da equipe de enfermagem no espaço da hemoterapia*

**DOI:** 10.1590/1980-220X-REEUSP-2024-0131pt

**Authors:** Francisco Gleidson de Azevedo Gonçalves, Silvia Teresa Carvalho de Araújo, Albert Lengruber de Azevedo, Priscila Brigolini Porfírio Ferreira, Danelia Gómez Torres, Fernanda de Nazaré Almeida Costa, Ariane da Silva Pires, Soraia do Socorro Furtado Bastos

**Affiliations:** 1 Universidade Federal do Rio de Janeiro, Escola de Enfermagem Anna Nery, Departamento de Enfermagem Médico-Cirúrgica, Rio de Janeiro, RJ, Brasil.; 2 Universidade Federal de Roraima, Centro de Ciências da Saúde, Boa Vista, Roraima, Brasil.; 3 Universidad Autonoma del Estado de México, Facultad de Enfermería, Departamento de Enfermería Médico-Quirúrgica, Toluca, Estado de México, México.; 4 Universidade do Estado do Rio de Janeiro, Faculdade de Enfermagem, Departamento de Enfermagem Médico-Cirúrgica, Rio de Janeiro, RJ, Brasil.

**Keywords:** Comportamento Espacial, Equipe de Assistência ao Paciente, Cuidados de Enfermagem, Comunicação não Verbal, Serviço de Hemoterapia, Transfusão de Sangue

## Abstract

**Objetivo:**

Analisar a influência dos fatores proxêmicos na comunicação e no cuidado prestado pelos profissionais de enfermagem durante o ato transfusional na hemoterapia.

**Método:**

Estudo descritivo, exploratório e qualitativo, com 25 profissionais de enfermagem de hospital especializado em doenças onco-hematológicas do Rio de Janeiro, a partir de roteiro sistematizado, registro individual dos fatores proxêmicos descritos por Edward Hall e entrevista situacional gravada. A análise considerou o conteúdo temático dos dados e utilizou o programa *SketchUp 3D Modeling Software Review* para demonstrar visualmente o mapeamento comportamental da interação dos profissionais de enfermagem com os clientes durante o cuidado.

**Resultados:**

As distâncias íntimas, pessoal, social e pública traduziram fatores proxêmicos, como os movimentos repetidos de ombros, pescoço e cabeça, que influenciaram positivamente o cuidado de enfermagem na hemoterapia, favorecendo a identificação das manifestações não verbais e verbais da equipe na interação com o cliente.

**Conclusão:**

A comunicação não verbal proxêmica deve ser resultante da consciência sobre disposição do espaço físico, das ações imediatas de cuidado, da pertinente e apropriada proximidade, da vigilância contínua visual e do reconhecimento manifesto das expressões traduzidas por interesse, respeito e zelo.

## INTRODUÇÃO

A comunicação proxêmica refere-se ao estudo social do espaço pessoal do ser humano no campo interacional, sendo determinada pelas distâncias que as pessoas mantêm entre si^(1)^. Seu reconhecimento pode se dar pela visão, audição, olfato e tato, além de variar muito entre as culturas e processos inerentes à construção do homem, à aculturação^(1)^, a situações e fenômenos, como a percepção, características fixas, semifixas e informais do espaço, e às reações e mudanças de comportamento^(2)^.

As características fixas dizem respeito aos edifícios, paredes, estruturas de aço, madeira, concreto, pontes e à organização das cidades; as características semifixas refere-se à disposição dos móveis de um cômodo, à utilização de adornos ou a qualquer comportamento do indivíduo que possa sugerir acolhimento, rejeição e fuga, cujas características podem ou não facilitar relacionamentos sociais; e as características informais são criadas pelos indivíduos para favorecer o estabelecimento de diferentes interações, como a distância íntima, que não ultrapassa 45 cm, a pessoal, de 46 cm até 120 cm, a social, de 120 cm até 370 cm, e a pública, acima de 370 cm^(1)^.

No cuidado de enfermagem, estar perto, manter interesse, prontidão e vínculo pode, intencionalmente ou não, expressar habilidades e competências que influenciam a relação com o outro. Mesmo que a comunicação proxêmica pareça ser uma barreira para muitos profissionais, expressões de dor, franzir da fronte, viradas de rosto ou até um gemido podem indicar necessidade de agir no espaço pessoal e territorial, instituir uma avaliação contínua, pensar a comunicação verbal e não verbal^(3)^, e discutir, ou mesmo elaborar, um plano de intervenções que considere o espaço onde os interlocutores se encontram presentes^(4)^.

Em cenários como a hemoterapia, diferente de outros ocupados por profissionais de enfermagem, o cuidado tende a se desenvolver sob duas perspectivas: o cuidado direto, que demanda a participação do doador, a coleta de dados, o momento da doação, o ato transfusional, ou mesmo um transplante de células sanguíneas vivas; e o cuidado indireto, que se aplica à bolsa de hemocomponente e se reverte à identificação, armazenamento, fracionamento, testagem, transporte e seu descarte adequado^(5,6)^.

Considerando que a comunicação deve ser entendida de modo dinâmico, o cuidado direto, mais precisamente o ato transfusional, exige constante aproximação entre as pessoas, escutas e olhares atentos e qualificados, por envolver a realização de procedimentos complementares e complexos, como a prescrição médica, a instalação do hemocomponente no receptor e sua monitorização, a identificação de pontos críticos, como a coleta da amostra, a decisão médica de transfundir ou não, bem como a administração do hemocomponente à beira do leito, possuindo, portanto, início, meio e fim^(7,8)^.

A experiência vivenciada em pesquisas relacionadas aos sentidos corporais com o intuito de compreender a comunicação proxêmica, a busca de fatores que influenciam e/ou interferem na percepção individual, a investigação de comportamentos e a interação com o cliente e equipe de saúde^(2,3)^, assim como as lacunas do conhecimento na área da hemoterapia sobre o mapeamento comportamental, contribuíram para delimitar a seguinte questão norteadora: que fatores proxêmicos determinam a comunicação dos profissionais de enfermagem durante o ato transfusional? De tal modo, o objetivo proposto foi analisar a influência dos fatores proxêmicos na comunicação e no cuidado prestado pelos profissionais de enfermagem durante o ato transfusional na hemoterapia.

## MÉTODO

### Desenho do Estudo

Estudo de natureza qualitativa, exploratória, descritiva, ancorado no referencial teórico de Edward T. Hall sobre comunicação proxêmica e mapeamento comportamental^(1)^.

### Local do Estudo

O cenário foi um hemocentro vinculado à secretaria estadual de saúde localizado na região Sudeste do Brasil. A escolha se deu porque esse é um serviço especializado, referência em hematologia e hemoterapia, que funciona todos os dias da semana, durante 24 horas, em regime ambulatorial e hospitalar, cuja missão é oferecer uma assistência de qualidade à população.

### Participantes e Critérios de Seleção

Participaram do estudo 25 profissionais de enfermagem do serviço de hemoterapia, que possuíam experiência mínima de seis meses e estavam inseridos na escala de pessoal de enfermagem do período diurno. Foram excluídos aqueles que não participaram dos dois momentos da coleta (observação e entrevista) e que se encontravam impedidos de realizar a assistência no momento da coleta (licença ou restrição médica).

### Procedimento Para Coleta de Dados

Para a coleta de dados, inicialmente, realizaram-se visita ao serviço e contato com a gerência de enfermagem e suas respectivas chefias setoriais, momento no qual foi esclarecido que a condução da pesquisa manteria assegurada e ininterrupta a assistência ao cliente e a garantia de que a relação do pesquisador com os participantes seria voltada para as ações e observações do comportamento proxêmico.

Em conformidade com o que foi acordado, deram-se a inserção e a familiarização do investigador no serviço mediante o encontro presencial com os profissionais de enfermagem, de forma privativa, por meio de um sistema de rodízio das 07:00 às 07:30. Seguiu-se com a explicação sobre o método do estudo e os itens considerados no roteiro de observação não participante. A seleção realizada pelo pesquisador considerou a técnica de conveniência, o interesse e a disponibilidade de cada participante.

A produção dos dados aconteceu entre abril e dezembro de 2022, quando, no primeiro momento, foi realizado o convite aos enfermeiros e técnicos de enfermagem para participar da pesquisa, seguindo-se da leitura e, posteriormente, da assinatura do Termo de Consentimento Livre e Esclarecido. A coleta foi realizada pelo pesquisador principal e por uma enfermeira que possui amplo conhecimento em pesquisa qualitativa na área da hematologia e hemoterapia, com ênfase na comunicação verbal e não verbal durante o ato transfusional. Para tal, utilizou-se um roteiro de observação não participante como instrumento.

Após o aceite, procedeu-se às observações da comunicação do profissional de enfermagem durante o ato transfusional. A observação não participante foi realizada com o objetivo de verificar o mapeamento comportamental da equipe de enfermagem durante o cuidado no ato transfusional. Foi utilizado um roteiro, criado a partir dos oito fatores proxêmicos propostos pela teoria de Hall, como postura-sexo, eixo sociófugo-sociópeto, fatores cinestésicos, comportamento de contato, código visual e volume da voz, e, por fim, o código térmico e olfativo. Essa etapa durou quatro horas por dia, e aconteceu por 90 dias, computando, ao todo, 360 horas.

Subsequentemente, foi realizada entrevista semiestruturada, composta por duas questões: quais os cuidados realizados durante o ato transfusional? Quais os cuidados relacionados às distâncias íntima, pessoal, social e pública? As perguntas foram feitas na instituição estudada, mediante disponibilidade dos participantes. Os pesquisadores gravaram as entrevistas individualmente em áudio, cuja duração média foi de 45 minutos.

No momento seguinte, que durou pouco mais de 360 horas, deu-se a reclusão do pesquisador, quando foram realizadas as transcrições das observações registradas em diário de campo, das entrevistas, bem como a leitura e a releitura do material produzido, o que possibilitou conhecer a interação do profissional de enfermagem com o cliente durante o ato transfusional e as distâncias mantidas no cuidado.

Foram considerados os critérios estabelecidos no *COnsolidated criteria for REporting Qualitative research* (COREQ)^(9)^ para manter o rigor do processo de organização dos dados.

### Análise e Tratamento dos Dados

Os dados foram organizados e preparados para a modalidade temática de análise de conteúdo^(10)^: leitura compreensiva e detalhada; análise do material; tratamento, compreensão e interpretação. Inicialmente, realizou-se a leitura criteriosa do material transcrito, com foco nas percepções dos profissionais sobre o fenômeno investigado. Em seguida, foram examinados os conteúdos dos discursos que apresentavam similaridades e diferenças, sendo reunidos em categorias de acordo com os objetivos da pesquisa. Para assegurar a confiabilidade dos dados, o pesquisador principal fez a transcrição das entrevistas, que foi revisada por um segundo investigador. Além disso, foram realizadas a dupla codificação e a verificação de erros, para garantir a legitimidade da criação da árvore de códigos.

Posteriormente, os códigos gerados foram agrupados por similaridades e diferenças na codificação axial, passando, ainda, por um refinamento na análise que deu origem à categoria central intitulada “Distâncias e proximidades traduzidas por sentidos corporais na hemoterapia”.

### Aspectos Éticos

Este estudo se atentou às normas nacionais e internacionais de ética em pesquisa envolvendo seres humanos, sendo aprovado, em 2020, pelo Comitê de Ética em Pesquisa, sob Parecer n^o^ 4.376.390. A pesquisa está em conformidade com a Resolução n^o^ 466, de 12 de dezembro de 2012, e foi utilizado o Termo de Consentimento Livre e Esclarecido como instrumento de consentimento. Garantiu-se o anonimato dos participantes, e seus depoimentos foram identificados como Téc. Enf. para a palavra “Técnico em Enfermagem” e Enf. para “Enfermeiro”, seguindo-se a ordem alfanumérica (1, 2, 3, até 18), conforme o código final apresentado (Téc. Enf.1, Téc. Enf.2, Téc. Enf.3 até Téc. Enf.18) e (Enf.1, Enf.2, Enf.3 até Enf.7).

## RESULTADOS

Os participantes do estudo foram sete enfermeiras e 18 técnicos de enfermagem (n = 25), em sua maioria mulheres (n = 22), casadas (n = 18), na faixa etária entre 30 e 45 anos (n = 18) e entre 45 e 55 anos (n = 7), com tempo de atuação superior a 13 anos no serviço de hemoterapia.

Considerando-se a ocupação da equipe de enfermagem no espaço físico da hemoterapia, identificaram-se os seguintes fatores proxêmicos: movimentos repetidos de ombros, pescoço e cabeça.

A partir do programa *Sketch Up 3D Modeling Software Review*, foi criada uma representação gráfica para ilustrar visualmente e, por meio de cores, o mapeamento comportamental resultante das observações realizadas na enfermaria geral e de precaução. Nela, o vermelho simboliza a distância íntima, que tem cerca de 45 cm de comprimento, e abrange desde o contato físico direto do profissional até o seu distanciamento do cliente. A tonalidade laranja varia de 45 cm a 1,20 m, indicando a distância pessoal que se mantém entre os indivíduos. A tonalidade amarela, que varia de 1,20 m a 3,60 m, simboliza a distância social. Por outro lado, a cor verde se estende de 3,60 m até os limites da visão ou audição, definindo a distância pública, conforme representado nas Figuras 1 e 2.

**Figura 1:** Representação gráfica da enfermaria geral. Rio de Janeiro, Brasil, 2023.

**Figura 2:** Representação gráfica da enfermaria de precaução. Rio de Janeiro, Brasil, 2023.

Nesse ambiente, há duas enfermarias para a hospitalização de clientes com desvios hematológicos, sendo uma delas composta por seis leitos e, a outra, por três, visando atender aqueles com algum tipo de precaução. Cada uma dessas enfermarias é separada por cortinas, e dispõe de cama/leito automatizada, mesa acessória, campainha - caso haja necessidade de solicitar a presença do profissional - e um biombo. A Figura 3 demostra o fluxo dos espaços da hemoterapia observados no momento da coleta de dados.

**Figura 3:** Representação gráfica do fluxo dos espaços da hemoterapia. Rio de Janeiro, Brasil, 2023.

O posto de enfermagem, por sua vez, fica longe das camas/leitos dos clientes, o que demanda a constante movimentação da equipe de enfermagem. A comunicação entre eles quase sempre se deu através de um painel de campainha que, ao ser acionado pelo cliente, indicava a necessidade de um profissional da equipe. Entre os mobiliários e insumos presentes nesse ambiente, observaram-se cadeiras, mesas, bancadas e materiais de consumo diário.

A posição em pé foi mencionada pelos participantes durante todos os cuidados relacionados à infusão e à retirada da bolsa de hemocomponentes, ou seja, desde a verificação de dados pessoais, como nome, matrícula, tipo sanguíneo e fator RH, até punção venosa periférica, retirada do acesso venoso periférico, bem como na alta hospitalar, conforme descrito:

*(...) eu fico em pé toda vez que vou administrar uma pré-medicação, para evitar a reação transfusional prévia; quando sou solicitada para retirar dúvidas; quando a bolsa de hemocomponente para de gotejar; quando é preciso puncionar um novo acesso; para visualizar a bolsa; quando tenho que manusear um cateter venoso profundo em femoral (Téc. Enf. 03).*

Já a posição sentada foi identificada em dois momentos. O primeiro foi durante a execução de cuidados burocráticos, como realizar evolução de enfermagem, atender telefonemas, conferir prescrições, entre outros. O segundo foi ao realizar algum tipo de cuidado sensível, como aproximar-se dos clientes para conversar ou mesmo esclarecer alguma dúvida acerca do processo transfusional, conforme compartilhado:

*(...) eu fico sentada quando estou realizando a evolução de enfermagem ou quando o cliente pergunta porque a bolsa está demorando muito; alguns deles gostam de conversar, e, nesse caso, eu paro tudo e puxo a cadeira para dar uma maior atenção (...) (Téc. Enf. 07).*

*Quando fico no posto de enfermagem realizando o acompanhamento da bolsa, atendendo ao telefone para conferir as solicitações dos andares, as prescrições médicas, verificar tudo e protocolar no livro, no mapa transfusional (Enf. 01).*

No que tange às distâncias preservadas entre a equipe de enfermagem e os clientes, a distância íntima ganhou destaque. Ela foi evidenciada pelos participantes durante a instalação de bolsa de hemocomponentes, verificação de sinais vitais, realização de punção venosa periférica, retirada de acessos e/ou mesmo na alta hospitalar, conforme apontado:

*Eu me aproximo toda vez que vou verificar os sinais vitais do cliente, a sua temperatura, para instalar uma transfusão de hemácias, um plasma ou até mesmo um concentrado de plaquetas, independente de qual seja o componente (Téc. Enf. 02).*

*(...) eu me aproximo mais do cliente quando tenho que conectar a bolsa de sangue; quando eu converso sobre o que ele pode sentir diante de uma reação; ou mesmo para verificar se ele já teve reação anterior (Téc. Enf. 14).*

Já a distância pessoal foi apresentada pelos participantes quando era necessário verificar os dados pessoais do cliente, conferir sua matrícula, tipo sanguíneo, fator RH e, também, durante conversa com o cliente. Cabe destacar que essa é uma distância na qual os interlocutores podem se tocar ou, até mesmo, manter um diálogo mais estreito sem que haja o toque.

*Eu já chego dando um “bom dia” e perguntando se ele está bem; busco verificar como ele está; como está o acesso venoso periférico; se não está fluindo bem a infusão; verifico se ele já teve alguma uma reação anterior à transfusão, o tipo sanguíneo, o fator RH, quando me identifico para o cliente (Enf. 07).*

*(...) quando eu abordo um cliente a primeira coisa que faço é perguntar o nome dele; apresentar-me; levanto o histórico de reações, os dados hemoterápicos em si como, por exemplo, as reações anteriores à transfusão; e, se eu for chamada, eu visualizo a bolsa; eu inspeciono tudo o que for necessário (Téc. Enf. 18).*

A distância social foi declarada pelos participantes em situações como, por exemplo, na primeira vez que o cliente recebeu uma bolsa de hemocomponentes, quando ele tocou a campainha o tempo todo, ou mesmo quando solicitou a presença de um profissional por causa do medo que sentia, conforme compartilhado:

*(...) tem cliente que fica muito receoso, nervoso e ansioso com a instalação do concentrado de plaquetas, principalmente aqueles que são a primeira ou a segunda transfusão. Eles solicitam a nossa permanência até se sentirem seguros. Nesses casos, eu busco me aproximar mais vezes naquela transfusão; fico atenta, vendo o rosto, vejo como ele está se comportando, principalmente se a transfusão está fluindo (Enf. 06).*

*(...) eu me aproximo todas as vezes que o cliente me chama (...) coloco ele o mais confortável possível; converso com ele; dou o máximo de atenção possível; pergunto se está tudo bem; me coloco disponível para conversar com ele (Téc. Enf. 13).*

A distância pública foi descrita pelos participantes em situações como atendimento às solicitações do cliente, ajustes na dosagem dos fatores de coagulação, recolhimento de pedidos em outros serviços da hemoterapia e/ou busca por prescrição de pré-medicação com hemoterapeuta, conforme depoimentos:

*(...) quando estou saindo do serviço, o cliente me chama, solicita algo, uma informação, que é mais comum. Eu sempre me aproximo dele, mesmo que não esteja perto fisicamente (Téc. Enf. 05).*

*(...) quando o cliente está em transfusão, eu tenho que ir lá verificar como ele está, como está o andamento do processo; tenho que ir também para retirar a bolsa e verificar os sinais vitais toda vez que termina (Enf. 04).*

## DISCUSSÃO

Em relação à descrição e à caracterização dos participantes, é importante observar que todos os enfermeiros eram especialistas em hemoterapia. Por outro lado, apenas três profissionais da equipe técnica de enfermagem tinham tal especialização. Suas práticas ocorrem de acordo com as leis, portarias e resoluções de diretoria colegiada da Agência Nacional de Vigilância Sanitária e do Ministério da Saúde, com procedimentos técnicos específicos que supervisionam e orientam a atividade dos enfermeiros e dos técnicos de enfermagem.

Profissionais experientes e conscientes, atentos às posturas, aos comportamentos e às atitudes, ao espaço físico, às ações de cuidado, à proximidade intensa, à vigilância visual contínua, e ao reconhecimento de expressões de interesse, respeito e zelo percebem a comunicação não verbal proxêmica. A pesquisa sinaliza que os médicos do sexo masculino têm voz alta porque ficam mais longe dos clientes e gastam mais tempo lendo os prontuários, enquanto que as médicas do sexo feminino têm clientes mais satisfeitos por causa de suas posturas corporais menos expansivas, vozes mais suaves e autotoque, transmitindo uma atmosfera mais atenta e médica^(4)^.

A observação participante e a análise das entrevistas permitiram identificar o mapeamento comportamental das cores que representam o distanciamento das formas de atendimento no ato transfusional. A cor vermelha simboliza a separação íntima, de cerca de 45 cm, entre o profissional e o cliente. A escala da cor laranja oscila entre 45 cm e 1,20 m, representando a distância que os indivíduos mantêm entre si. A distância social é representada pela medida amarela, que varia de 1,20 m a 3,60 m. A tonalidade verde simboliza a extensão pública de 3,60 m até os limites de visão ou audição^(1)^.

Durante a verificação de sinais vitais, punção venosa periférica, instalação de bolsas de hemocomponentes, retirada de acessos e, até mesmo, na alta hospitalar, por exemplo, a cor vermelha indica uma distância íntima. Os dados pessoais do cliente, como a matrícula, o tipo sanguíneo e o fator RH, são representados pela cor laranja para demonstrar a distância pessoal. A cor amarela simboliza o distanciamento social, que é visto em circunstâncias como na primeira vez que o cliente recebe uma bolsa de hemocomponentes, em que ele frequentemente toca a campainha ou mesmo solicita a presença de um profissional por medo. Já a cor verde representa o distanciamento social, que pode ser usado em situações como atender aos pedidos dos clientes, ajustar as dosagens dos fatores de coagulação, ou mesmo receber pedidos de outros serviços de hemoterapia.

Quando reconhecidas as distâncias mensuráveis no processo relacional, a comunicação proxêmica tende a se tornar uma ferramenta a mais para que os profissionais de enfermagem e de saúde complementem o cuidado, de forma a valorizar as relações interpessoais e promover saúde e bem-estar^(1)^. Assim, na dimensão comunicacional, o uso do espaço é representado por uma distância nos momentos da prática do cuidar na hemoterapia.

Ao observar a linguagem proxêmica, as relações interpessoais e a distância mantida na interação^(3,11)^, fica claro que o espaço íntimo é adotado pela maior parte dos participantes, representado por uma postura frente a frente que direciona o olhar para o cliente, observando, orientando e facilitando a relação interacional. Essa relação pode ser compreendida a partir do conjunto das observações que os indivíduos fazem do espaço e de como o usam e interpretam dentro do processo comunicativo. Já a linguagem proxêmica é influenciada por normas culturais, pelo contexto, pelos obstáculos espaciais, pelas relações entre os interlocutores e pelo grau de afinidade entre eles^(12)^.

No contexto da hemoterapia, a comunicação proxêmica representa uma ferramenta poderosa e facilitadora ao processo de interação entre os sujeitos, mas que carece ser explorada constantemente^(1)^. Isso exige treino e disposição. A observação atenta aos sinais não verbais possibilita uma percepção fidedigna da mensagem^(11)^. Para interpretá-la, é preciso saber lidar com a própria maneira de se comunicar. Sua efetividade aumenta quando se tem consciência da importância da linguagem corporal, principalmente a proximidade, a postura e o contato visual^(11)^.

A investigação dos comportamentos no ambiente hospitalar de alta complexidade da hemoterapia é um processo difícil, pois ora os cuidados se destinam aos componentes sanguíneos, ora ao cliente, o que pode tornar frágil sua operacionalização. Ademais, atitudes e gestos costumam ser difíceis de serem identificados e categorizados, uma vez que o atendimento ao cliente com produtos sanguíneos é longo e, dependendo da condição, constante, tornando-se um hábito ao longo da vida^(13)^.

O mapa de comportamento criado a partir das zonas de distância foi empregado para relacionar as distâncias entre indivíduos, enquanto que as cores foram empregadas para demonstrar as características proxêmicas da equipe de enfermagem envolvida nos procedimentos de hemoterapia. Isso oportuniza ampliar a possibilidade de observar e detectar os sinais verbais ou não verbais do cliente, contribuindo para uma comunicação e relações interpessoais mais efetivas entre os sujeitos da interação, uma vez que permite analisar e modificar determinados comportamentos ou posturas em abordagens e áreas pessoais^(1,2)^.

Neste cenário, a distância íntima é mais evidente durante a instalação de um componente sanguíneo, na preparação da pele para a punção venosa e na remoção dos acessos. A assistência oferecida requer uma proximidade máxima entre o profissional de enfermagem em hemoterapia e o cliente, cujos laços podem se estreitar, dado que as interações acontecem continuamente, ininterruptamente, durante as 24 horas do plantão, mês após mês, ano após ano, resultando em uma aproximação intensa, devido ao tempo de permanência e ao tratamento prolongado^(11,12)^.

A distância pessoal ocorre quando os profissionais abordam os clientes a uma certa distância, perguntam o seu nome, apresentam-se, identificam o histórico de reações anteriores à transfusão, visualizam a bolsa e inspecionam a transfusão^(13)^.

Em relação à distância social, as interações são determinadas pelo vínculo estabelecido entre as pessoas, pelo sentimento existente e pela forma de cuidado naquele instante. Na hemoterapia, a comunicação entre o enfermeiro e o cliente se destaca, a cada dia, pela proxemia, seja no momento de observação do ato transfusional à distância, do olhar mais aguçado em busca de reações transfusionais, ou no período de espera dos dez primeiros minutos após instalação do hemocomponente, sentado no posto de enfermagem^(14,15)^.

Na distância pública, a visão é prejudicada, assim como a nitidez das imagens. O tom de voz é elevado, o que pode ser comparado a um comportamento de fuga^(14,16)^. Portanto, é um grande desafio identificar métodos que permitam identificar comportamentos proxêmicos que se manifestam na realidade diária da equipe de enfermagem. Os sinais não verbais fornecem pistas sobre a interação das pessoas com os ambientes, que não poderiam ser obtidos por outros métodos de pesquisa^(1,14,16)^.

Nesta investigação, a distância pública se modificou para íntima quando o profissional se encontrava atento, nos primeiros minutos de transfusão, e quando o cliente se despedia de longe. Desse modo, a distância pública é estabelecida durante todo o processo transfusional, e a íntima, ao finalizá-lo, com a verificação dos sinais vitais após transfusão.

É importante ressaltar que o contexto onde as pessoas se comunicam muitas vezes influencia a aproximação ou o distanciamento dos corpos e que tanto a regularidade quanto o conteúdo das mensagens são afetados por diversos elementos do ambiente^(1)^. Esse ambiente afeta o comportamento, mas também pode alterá-lo e provocar determinadas reações. Quando se tem conhecimento do local, pode-se propositalmente utilizá-lo para alcançar as respostas pretendidas. É evidente que a proximidade possibilita a obtenção de mais informações sobre o outro indivíduo^(1)^.

A disposição dos espaços fixos e semifixos ocupados pelos clientes impacta o comportamento proxêmico do profissional e o seu desenvolvimento, principalmente quando há, *a priori,* conhecimento e consciência do conceito e influência perceptiva.

Portanto, o tamanho e a disposição dos objetos e móveis, a iluminação apropriada, a cor do teto e a temperatura do ambiente afetam o percurso e a interação visual e física entre os indivíduos. Se um ambiente mais acessível pode aumentar a frequência de interações, pequenas alterações no espaço também podem facilitar e favorecer a proxemia de aproximação^(1,17)^, expandindo o acesso e a interação.

Assim, a atenção a esses elementos afeta a comunicação, definindo habilidades e competências na interação com o próximo^(18)^. Manter proximidade, interesse, disponibilidade e conexão facilita a expressão de emoções, necessidades e avaliação durante a interação^(19,20)^.

Portanto, como profissão ligada à saúde, a enfermagem deve também se preocupar com o padrão de comunicação proxêmica no ambiente de atendimento no contexto da hemoterapia^(18)^. A habilidade de proporcionar ao cliente a chance de uma vida mais digna e compreensível requer o conhecimento da linguagem corporal e a compreensão de sua posição no contexto terapêutico^(2,16)^.

É importante destacar que as ações de cuidado foram predominantemente marcadas pelos gestos dos profissionais em ambientes íntimos, pessoais, sociais e públicos. Além disso, a verbalização da equipe de enfermagem na hemoterapia provocou variações no uso e na extensão dos sentidos corporais. Portanto, para que seja possível pensar, debater, criar e implementar estratégias de intervenção ao cliente, é crucial que a equipe de enfermagem em hemoterapia compreenda primeiramente o espaço como um dos elementos que cercam os interlocutores, podendo influenciar, ocasionalmente, o grau e o tipo de interação que se dá^(18,21)^.

Por se tratar de uma temática que aborda a comunicação proxêmica como forma de comunicação não verbal, o estudo teve como limitação a COVID-19. Não foi possível verificar toda a proxemia em sua integralidade, pois a utilização da máscara, necessária ao momento pandêmico, influenciou algumas observações das expressões faciais e corporais dos participantes nas interações. Nesse sentido, o uso desse equipamento de proteção trouxe algumas dificuldades na interação, como embaraço para se comunicar oralmente e dificuldade na percepção das expressões faciais. Clientes e enfermeiros não conseguiam se comunicar bem e falavam mais alto, além de não instituírem a leitura labial e o reconhecimento de outras pistas visuais na face.

Recomenda-se o desenvolvimento de estudos multicêntricos em outras regiões do país e em outros hemocentros, ainda carentes desses dados, com abordagem quantitativa, que poderiam elucidar melhor as distâncias e proximidades nas relações entre cliente e profissional durante o cuidado de enfermagem. Soma-se a isso o fato de o Brasil dispor de poucas pesquisas cujo interesse é a comunicação proxêmica na hemoterapia.

Acredita-se que os resultados deste estudo possam contribuir para o aperfeiçoamento da análise do comportamento proxêmico do enfermeiro, bem como para que outros hemocentros e instituições considerem a incorporação desses achados para qualificar e beneficiar os clientes.

## CONCLUSÃO

Os resultados revelam que a comunicação não verbal proxêmica é influenciada pelo espaço físico, ações de cuidado, relação de proximidade, vigilância visual contínua, reconhecimento de expressões faciais, respeito e zelo. Como consequência, para que um cuidado de qualidade seja ofertado, é essencial que os profissionais estejam atentos às posturas, comportamentos e atitudes de seus clientes. Esse tipo de comunicação demanda atenção constante, escuta qualificada e olhares atentos, sendo um processo que deve ser compreendido de forma dinâmica.

A identificação das expressões não verbais e verbais da equipe de enfermagem da hemoterapia na interação com o cliente foi facilitada pelo mapeamento comportamental, que utilizou os sentidos corporais para identificar sinais e expressões não verbais. Ele possibilitou a verificação de como ambos os participantes podem se beneficiar ou não nas interações e no atendimento no âmbito da hemoterapia. O contexto da hemoterapia é singular, e o comportamento dos profissionais de enfermagem requer o desenvolvimento de uma ferramenta para avaliar as interações no atendimento.

Constatou-se que o comportamento proxêmico é influenciado pelo espaço físico e que as medidas tomadas pelos profissionais na hemoterapia influenciam o mapeamento observado nas interações e no cuidado prestado ao cliente. No estudo, a equipe de enfermagem na hemoterapia demonstrou um bom vínculo com o cliente, demonstrando interesse, respeito e cuidado, evidenciado através de uma proximidade intensa, através de monitoramento visual constante, principalmente após instalação de um hemocomponente.

O comportamento proxêmico, que faz parte da linguagem não verbal, costuma ser misterioso no processo de comunicação. Normalmente, as interações da equipe de saúde com os clientes durante a hemoterapia se deram em todas as distâncias sugeridas por Hall, com predomínio da distância íntima, seguida pela distância pessoal, distância social e distância pública, todas estabelecidas durante a assistência de enfermagem na hemoterapia. Os achados ressaltaram que o comportamento proxêmico e a análise comportamental auxiliaram na identificação de expressões não verbais e verbais. Além disso, a interação da equipe de enfermagem da hemoterapia com os clientes influenciou o uso dos sentidos corporais para identificar sinais e expressões não verbais.

Ainda existem estudos iniciais sobre a comunicação proxêmica entre a equipe de enfermagem e o cliente durante a hemoterapia. Portanto, considera-se que este estudo pode servir como um ponto inicial para futuras pesquisas, especialmente para implementar ações da equipe e (re)examinar a comunicação com esse público.
